# Identifying a locus in super-enhancer and its resident NFE2L1/MAFG as transcriptional factors that drive PD-L1 expression and immune evasion

**DOI:** 10.1038/s41389-023-00500-3

**Published:** 2023-11-20

**Authors:** Conglin Shi, Liuting Chen, Hui Pi, Henglu Cui, Chenyang Fan, Fangzheng Tan, Xuanhao Qu, Rong Sun, Fengbo Zhao, Yihua Song, Yuanyuan Wu, Miaomiao Chen, Wenkai Ni, Lishuai Qu, Renfang Mao, Yihui Fan

**Affiliations:** 1 Department of Pathogenic Biology, School of Medicine, Nantong University, Nantong, 226001 China; 2 Laboratory of Medical Science, School of Medicine, Nantong University, Nantong, 226001 China; 3 Department of Pathophysiology, School of Medicine, Nantong University, Nantong, 226001 China; 4 Shanghai Chongming Center for Disease Control and Prevention, Shanghai, 202150 China; 5 Department of Stomatology, Affiliated Hospital of Nantong University, Medical School of Nantong University, Nantong, 226001 China; 6 Department of Gastroenterology, Affiliated Hospital of Nantong University, Medical School of Nantong University, Nantong, 226001 China

**Keywords:** Cancer genetics, Cancer genetics

## Abstract

Although the transcriptional regulation of the programmed death ligand 1 (PD-L1) promoter has been extensively studied, the transcription factor residing in the PD-L1 super-enhancer has not been comprehensively explored. Through saturated CRISPR-Cas9 screening of the core region of the PD-L1 super-enhancer, we have identified a crucial genetic locus, referred to as locus 22, which is essential for PD-L1 expression. Locus 22 is a potential binding site for NFE2:MAF transcription factors. Although genetic silencing of NRF2 (NFE2L2) did not result in a reduction of PD-L1 expression, further analysis reveals that MAFG and NFE2L1 (NRF1) play a critical role in the expression of PD-L1. Importantly, lipopolysaccharides (LPS) as the major component of intratumoral bacteria could greatly induce PD-L1 expression, which is dependent on the PD-L1 super-enhancer, locus 22, and NFE2L1/MAFG. Mechanistically, genetic modification of locus 22 and silencing of MAFG greatly reduce BRD4 binding and loop formation but have minimal effects on H3K27Ac modification. Unlike control cells, cells with genetic modification of locus 22 and silencing of NFE2L1/MAFG failed to escape T cell-mediated killing. In breast cancer, the expression of MAFG is positively correlated with the expression of PD-L1. Taken together, our findings demonstrate the critical role of locus 22 and its associated transcription factor NFE2L1/MAFG in super-enhancer– and LPS-induced PD-L1 expression. Our findings provide new insight into understanding the regulation of PD-L1 transcription and intratumoral bacteria-mediated immune evasion.

## Introduction

Programmed death ligand 1 (PD-L1) and programmed death 1 ligand 2 (PD-L2) are ligands that bind to the receptor PD-1 to restrict the activated T lymphocytes. They frequently express on tumors and cause tumor cells to escape killing by T lymphocytes during tumor development. Thus, therapies blocking the interaction between PD-L1 (L2) and PD-1 through either anti-PD-L1 or anti-PD-1 antibodies can remove the restriction on T cells and fully activate the antitumor immune response, which already substantially improve the outcomes of cancer patients [1–3]. However, the percentage of patients who benefit from these therapies is ~20–40%, and fewer patients will have long-term disease remission [4–6]. Therefore, developing methods to identify the benefiting patients, overcoming the primary and acquired resistance, avoiding immune-related adverse effects, and establishing novel combinational therapies for nonresponsive patients are all critical to further substantially potentiate therapeutic effects. Nevertheless, these challenging tasks are heavily dependent on a better understanding of the molecular regulation of PD-L1 and PD-L2. The abnormal production of PD-L1 in tumor cells is caused by dysregulation of many mechanisms from gene transcription to extracellular presentation [2, 7]. Although abnormal regulation at different layers contributes to the high expression of PD-L1 in tumor cells, the fundamental question is how tumor cells initiate the transcription of PD-L1 that should be turned off normally.

PD-L1 is encoded by CD274 located at chr9p24.1, and the transcriptional regulation of its promoter is extensively investigated. For example, in the promoter region of PD-L1, several NF-κB binding sites induce PD-L1 expression under different stimulations [8, 9]. The interferon family, including interferon (IFN)-α, IFN-β, and IFN-γ, is a strong inducer for PD-L1 expression in cancer. Upon interferon binding to receptors, interferons promote transcription factors including STAT1, STAT2, STAT3, and IRF1 to bind to PD-L1 and PD-L2 promoters and induce the expression of PD-L1 and PD-L2 [10]. Although a group of common inflammation-associated transcription factors that bind to the PD-L1 promoter have been reported, it is still largely undetermined how PD-L1 transcription is uniquely orchestrated between the promoter and other regulatory DNA elements. Recently, we found a super-enhancer (PD-L1L2-SE) between PD-L1 and PD-L2 encoding genes, which can induce the synchronous expression of PD-L1 and PD-L2 [11, 12]. However, the transcription factors that bind to PD-L1L2-SE are largely unexplored.

CNC (Cap’n’collar) transcription factors including NFE2L1 and NFE2L2 possess CNC domains and bZIP motifs that are conserved in many species [13]. Phenotypes from knockout mice demonstrate nonredundant roles of NFE2L1 in embryonic development and NFE2L2 in response to oxidative stress [14]. In cancer, NFE2L2 promotes aggressive tumorigenesis and confers therapeutic resistance via metabolic reprogramming and increased antioxidant capacity [15]. NFE2L1 plays a critical role in therapeutic resistance to proteasome inhibitors [16]. Because CNC transcription factors cannot bind to DNA as monomers, sMafs (small Maf proteins), including MafG, MafF, and MafK, are indispensable partners that are required by CNC proteins to exert their function [17]. Although the function of NFE2L1/2 and its binding partners (MafG, MafF, and MafK) to form heterodimers in cancer has been extensively studied, their distinguishing roles in the regulation of PD-L1 and immune evasion are poorly understood. Previous reports have identified that NFE2L2 is a critical transcriptional factor in the regulation of IFN-γ– and ultraviolet radiation-induced PD-L1 expression [18, 19]. However, it is inconclusive whether NFE2L2 also regulates PD-L1 via the super-enhancer, and transcriptional factors that reside on the PD-L1L2-SE super-enhancer are undetermined.

Recently, several studies demonstrated that the intratumoral microbiome was frequently observed in cancers and that more than 60% of breast cancer tissues are positive for bacterial DNA [20, 21]. However, the function of intratumoral bacteria in cancer development is unexplored. Lipopolysaccharides (LPS) are the major outer surface membrane components present in gram-negative bacteria and also frequently exist in cancers, but the role of intratumoral LPS is largely undetermined. Previous studies indicate that LPS are inducers of PD-L1 transcription via activation of NF-κB, which binds to the PD-L1 promoter [22–24]. However, it is inconclusive whether LPS-induced PD-L1 is dependent on the PD-L1L2-SE super-enhancer and, if so, what the downstream transcription factors are. Here, we identified a locus and its resident transcription factor, NFE2L1/MAFG, which are essential for persistent or LPS-induced PD-L1 expression. Our findings provide new insights into understanding the transcriptional regulation of PD-L1 between the promoter and the PD-L1L2-SE super-enhancer.

## Results

### CRISPR-Cas9 screening identifies a genetic locus in the super-enhancer region that is critical for PD-L1 expression

**Fig. 1:**
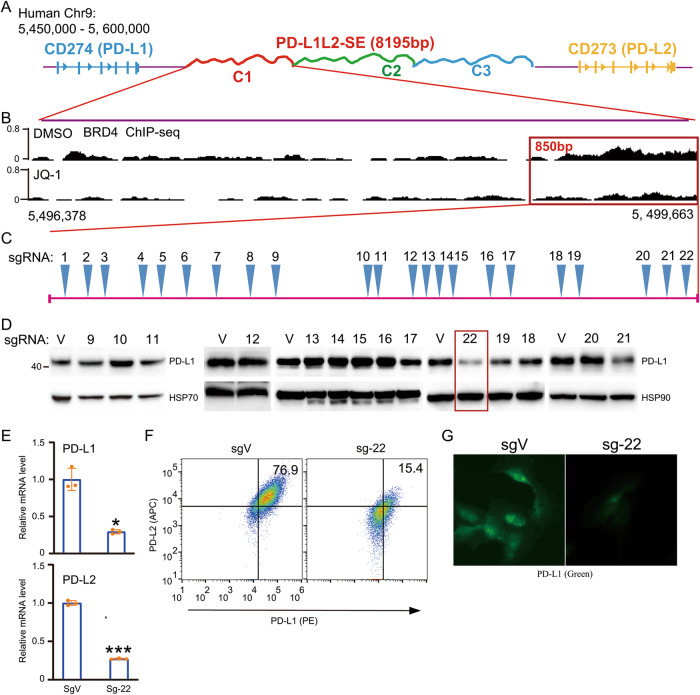
Tiled sgRNAs identify a locus that critical for super-enhancer-induced PD-L1 expression. **A** Schematic representation of the genomic locations of *CD274*, *CD273*, as well as the super-enhancer PD-L1L2-SE, which was divided into 3 elements (C1, C2, C3); **B** Precise analysis of BRD4 binding region between JQ1 and DMSO treated cells from Chr9:5,496,378 to Chr9:5,499,663. BRD4 ChIP-sequencing data was downloaded from GEO database: GSM2330549 and GSM2330551; **C** The location of a 850 bp DNA region in PD-L1L2-SE super-enhancer and saturated design of all potential specific sgRNAs; **D** SUM-159 cells were stably transfected with indicated sgRNAs. Then western blotting analysis shows the protein level of PD-L1 in each stably genetic modified cell lines. **E** Real-time PCR was performed to analyze the mRNA level of PD-L1 and PD-L2 in sgVector and Sg-22 cells. **F** The surface expression of PD-L1 (PE) and PD-L2 (APC) in sgVector and sg-22 cells were determined by FACS. **G** Immunofluorescence was performed to analyze the distribution and expression of PD-L1 (Green) in sgVector and sg-22 cells. Hoechst was used as nuclear staining. Data in (**D**) and (**G**) are representative of two independent experiments. Data in (**E**) and (**F**) are representative of three independent experiments. *p < 0.05; **p < 0.01, ***p < 0.001.

Previously, we identified a super-enhancer and its core region that are essential for the constitutive expression of PD-L1 and PD-L2 in cancer cells (Fig. 1A) [11, 12]. Here, we further analyzed the core region using BRD4 ChIP-seq data with or without JQ-1 treatment (Fig. 1B). The data reveal a DNA region of ~850 bp that is highly enriched with BRD4 and that the binding of BRD4 was greatly reduced by JQ-1 treatment (Fig. 1B). To identify an important locus that is essential for the super-enhancer-induced expression of PD-L1 and PD-L2, we designed all potentially specific guide RNAs (gRNAs), including 22 sgRNAs (Fig. 1C). Next, we established genetically modified cell lines for each sgRNA in SUM-159 cells, which exhibit the constitutive expression of PD-L1 and PD-L2. We used WB to examine the expression of PD-L1 in each genetically modified cell line (Fig. 1D). We identified two loci (loci 19 and 22) that greatly reduce the expression of PD-L1 (Fig. 1D). In this paper, we focused on locus 22 (sg-22). To further confirm the effect of locus 22 on the expression of PD-L1 and PD-L2, we performed RT-PCR to examine the mRNA level of PD-L1 and PD-L2 in sg-22 cells (Fig. 1E). Compared with that in control cells, the expression of PD-L1 and PD-L2 was greatly reduced in sg-22 cells. Consistently, flow cytometry and immunofluorescence further confirmed the reduction of PD-L1 and PD-L2 in sg-22 cells (Fig. 1F, G). Our results demonstrate that genetic modification of locus 22 has a profound effect on the expression of PD-L1 and PD-L2.

### NFE2/MAF are potential transcription factors that recognize locus 22 and are important for PD-L1 expression

**Fig. 2:**
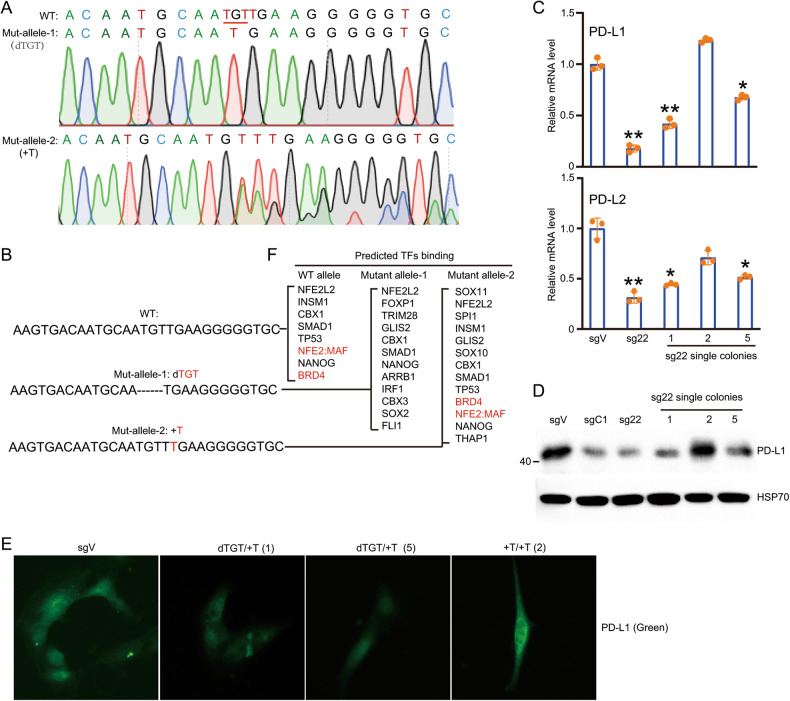
Precisely mapping the nucleotide acid in locus 22 and its resident transcriptional factors NFE2:MAF. **A** Cloning and direct sequencing of genetic modified locus 22 by T vectors. **B** DNA sequences of wild-type and two single cell colonies. One is deletion of TGT and another one is addition of T; **C** Three single cell colonies (1,2,5) were selected and expanded for experiments. Real-time PCR was performed to analyze the mRNA level of PD-L1 and PD-L2 in sgVector, pooled sg-22 and three single cell colonies separated from pooled sg-22; **D** Western blot was performed to analyze the protein level of PD-L1 in sgVector, sgC1, pooled sg-22 and three single cell colonies from pooled sg-22; **E** Immunofluorescence was used to examine the expression and distribution of PD-L1 in sgVector and three single cell colonies from pooled sg-22; **F** The potential transcription factors at 30 bp DNA region around locus 22 in wild-type and dTGT were analyzed by online programs AnimalTFDB (http://bioinfo.life.hust.edu.cn/AnimalTFDB). All potential transcription factors at each allele were listed. The binding of NFE2:MAF was disrupted after deletion of TGT. Data in (**C**) and (**D**) are representative of three independent experiments. Data in (**E**) are representative of two independent experiments. *p < 0.05; **p < 0.01, ***p < 0.001.

The aforementioned results demonstrate that genetic modification of locus 22 affects the expression of PD-L1 and PD-L2. To reveal the change in DNA sequence at locus 22, we cultured single cell colonies of locus 22 cells. The genomic DNA of locus 22 was amplified and cloned to T vector for sequencing. On the basis of the sequencing results, we found two types of genetic changes: one is the deletion of three TGT nucleotides, and another is the addition of a T nucleotide (Fig. 2A, B). We selected two colonies with the deletion of TGT (colonies 1 and 5), one colony with the addition of T (colony 2), and the pooled cells of locus 22. Compared with that in control cells, the expression of PD-L1 and PD-L2 was greatly reduced in the pooled cells and cells with the deletion of TGT but not the addition of T (Fig. 2C). To further confirm this result, we performed WB and immunofluorescence. As indicated, the deletion of TGT at locus 22 greatly reduces the expression of PD-L1, but the addition of T at locus 22 has a minimal effect (Fig. 2D, E). These results demonstrate that the deletion of TGT at locus 22 greatly reduces the expression of PD-L1. To uncover the change of potential transcription factors before and after genetic modification at locus 22, we analyzed the 30-bp DNA sequence around locus 22. As indicated, NFE2:MAF binds to wild-type allele, and the deletion of TGT but not the addition of T disrupts the binding of NFE2:MAF (Fig. 2F). These results suggest that disrupting the binding of NFE2:MAF potentially reduces the expression of PD-L1.

### LPS strongly induces PD-L1 expression, and LPS-induced PD-L1 is dependent on the super-enhancer and locus 22

**Fig. 3:**
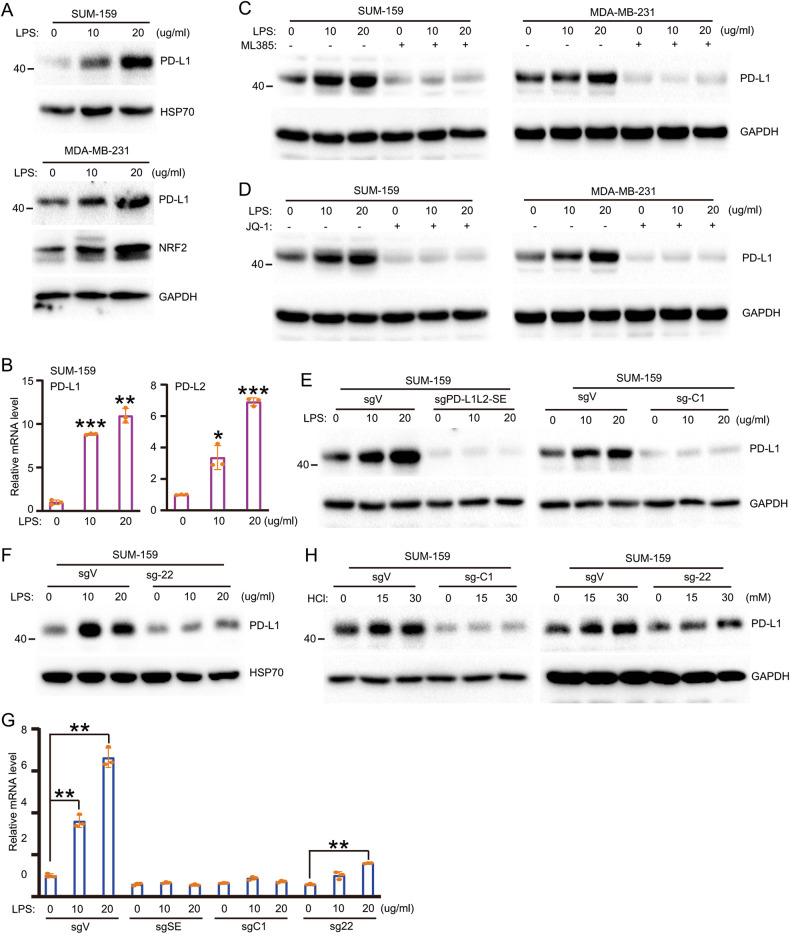
LPS strongly induces PD-L1 expression via super-enhancer and its core DNA region. **A** SUM-159 cells and MDA-MB-231 cells were treated with indicated concentration of LPS for 24 h and western blot analysis was performed to examine the expression of PD-L1; **B** Relative mRNA level of PD-L1 and PD-L2 was examined by RT-PCR in LPS treated SUM-159 cells; **C** ML385 was used to pre-treat cells for 6 h, then LPS was added for treatment at indicated doses for 24 h. The expression of PD-L1 was examined by western blot; **D** JQ-1 was used to pre-treat cells for 6 h, then LPS was added for treatment at indicated doses for 24 h. The expression of PD-L1 was examined by western blot; **E** Western blotting was used to quantify the protein level of PD-L1 in LPS treated sgVector, sgPD-L1L2-SE and sgPD-L1L2-SE-C1 cells; **F** Western blotting was used to quantify the protein level of PD-L1 in LPS treated sgVector and sg-22 cells; **G** Relative mRNA level of PD-L1 was examined by RT-PCR in LPS treated sgV, sgSE, sgC1 and sg22 cells; **H** HCl was used to treat cell and the protein level of PD-L1 in sgV, sg-C1 and sg-22 was determined by WB. Data in (**A**–**F**) are representative of three independent experiments. Data in (**G**) and (**H**) are representative of two independent experiments. *p < 0.05; **p < 0.01, ***p < 0.001.

The existence of the intratumoral microbiome is a common phenomenon, and the function of bacterial LPS has been associated with NFE2:MAF [20, 21]. Thus, we examined the effect of LPS on PD-L1 and whether this effect is dependent on the super-enhancer and NFE2:MAF. In SUM-159 and MDA-MB-231 cells, LPS treatment strongly induces the expression of PD-L1 (Fig. 3A). RT-PCR further confirmed the increased PD-L1 transcription after LPS treatment (Fig. 3B). Treatment with ML385, the inhibitor of NFE2:MAF, almost completely inhibits LPS-induced PD-L1 expression (Fig. 3C). The super-enhancer inhibitor (JQ-1) also completely inhibits LPS-induced PD-L1 expression (Fig. 3D). These results indicate the critical role of the super-enhancer and NFE2:MAF complex on LPS-induced PD-L1 expression. To support this conclusion, we treated control and PD-L1L2-SE-deficient cells with LPS. Again, LPS strongly induce PD-L1 expression in control cells but not in PD-L1L2-SE-deficient cells (Fig. 3E). Consistently, knockout of the core C1 region of the PD-L1L2-SE super-enhancer (PD-L1L2-SE-C1) also completely blocks LPS-induced PD-L1 expression (Fig. 3E). Genetic modification of locus 22 also strongly inhibits LPS-induced PD-L1 expression (Fig. 3F, G). But unlike deletion of SE- or C1, which totally block the LPS-induced PD-L1 expression, deletion of locus 22 still shows slightly induction of PD-L1 upon LPS treatment. To further explore the role of PD-L1L2-SE in the regulation of PD-L1, we used HCl to induce acidic tumor microenvironment. As reported, HCl treatment also induces the expression of PD-L1 [25] (Fig. 3H). However, HCl failed to induce the expression of PD-L1 in C1 deficient cells (Fig. 3H). In locus 22 deficient cell, similar to LPS, HCl-induced PD-L1 was greatly reduced (Fig. 3H). Taken together, our results demonstrate that LPS and HCl are strong inducers of PD-L1 expression and that the effect of LPS and HCl on PD-L1 is dependent on the PD-L1L2-SE super-enhancer, C1 region and locus 22.

### NEF2L2 (NRF2) is not required for super-enhancer- and LPS-induced PD-L1 expression

**Fig. 4:**
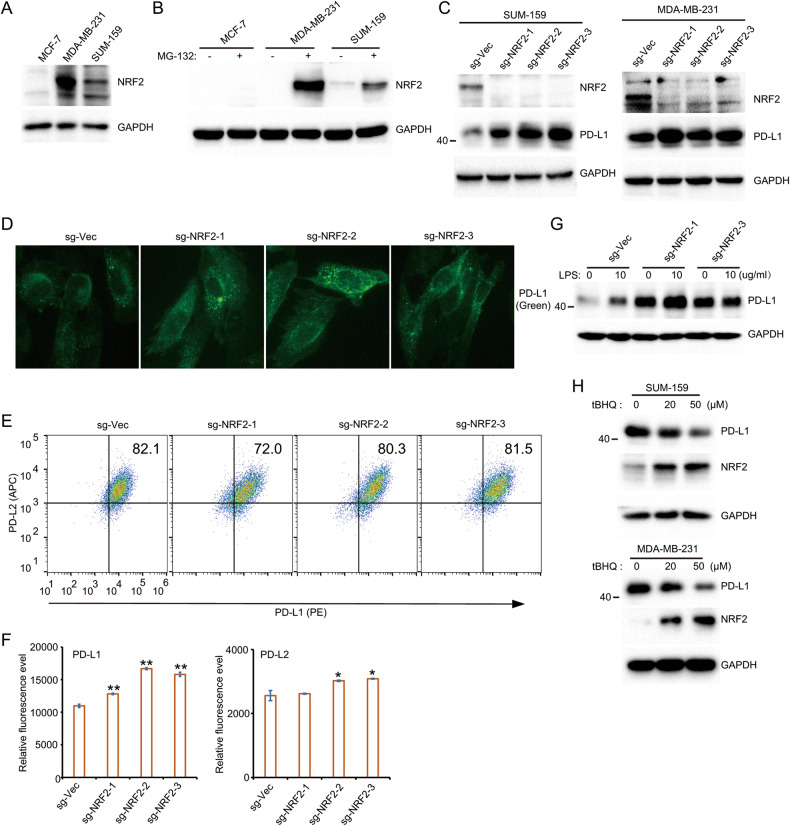
NRF2 (NFE2L2) is not required for super-enhancer- and LPS-induced PD-L1 expression. **A** Western blot analysis was performed to detect the expression of NRF2 in MCF-7, SUM-159 and MDA-MB-231 cells; **B** MCF-7, SUM-159 and MDA-MB-231 cells were treated with or without MG132 and the protein level of NRF2 was determined by WB; **C** Three sgRNAs targeted on NRF2 was stably introduced in SUM-159 and MDA-MB-231 cells. The protein level of NRF2 and PD-L1 was examined in control and NRF2-deficient cells; **D** Immunofluorescence assays were used to examine the expression of PD-L1 in NRF2-deficient cells; **E** Representative FACS images to indicate the expression of PD-L1 (PE) and PD-L2 (APC) in NRF2-deficient and control SUM-159 cells. **F** Summary of the mean fluorescence intensity from (**E**); **G** Control and NRF2-deficient SUM-159 cells were treated with LPS at indicated doses. The protein level of PD-L1 in these cells was analyzed by Western blot. **H** Cells were treated by tBHQ for indicated doses and the protein level of PD-L1 and NRF2 was examined by WB. Data in (**A**–**C** and **G**) are representative of three independent experiments. Data in (**D**, **E** and **H**) are representative of two independent experiments. *p < 0.05; **p < 0.01, ***p < 0.001.

Our results suggest the critical role of NFE2:MAF transcription factors in super-enhancer- and LPS-induced PD-L1 expression. Thus, we determined the role of NFE2L2 (NRF2), the most studied transcription factor in the NFE2:MAF family, in the regulation of PD-L1. Consistent with the high expression of PD-L1 in SUM-159 and MDA-MB-231 cells, the expression of NRF2 is also high in SUM-159 and MDA-MB-231 cells (Fig. 4A). MG-132 treatment blocks NRF2 degradation and significantly increases NRF2 accumulation in SUM-159 and MDA-MB-231 cells but not in MCF-7 cells (Fig. 4B). To explore the role of NRF2 in the regulation of PD-L1, we established NRF2 knockout cells using three individual sgRNAs. As indicated, all three sgRNAs almost completely disrupt the expression of NRF2 (Fig. 4C). However, surprisingly, knockout NRF2 in cells does not reduce the expression of PD-L1 (Fig. 4C). Furthermore, immunofluorescence and flow cytometry demonstrated the slightly increased expression of PD-L1 in NRF2-deficient cells (Fig. 4D–F). These results demonstrate that NRF2 is not required for super-enhancer-induced PD-L1 expression. Next, we treated cells with LPS in control and NRF2-deficient cells. As indicated, LPS strongly induce the expression of PD-L1 in control cells. NRF2-deficient cells exhibit high expression of PD-L1, and the expression of PD-L1 was further upregulated upon LPS treatment (Fig. 4G). To further validate the negative regulatory role of NRF2 on PD-L1, we used tBHQ (Tert-butylhydroquinone), which is a known inducer of NRF2. As expected, tBHQ increases the level of NRF2. The level of PD-L1 was greatly reduced upon tBHQ treatment, which supports the inhibitory role of NRF2 on PD-L1 expression in SUM-159 cells (Fig. 4H). Taken together, our results demonstrate that NRF2 is not a positive regulator in super-enhancer- and LPS-derived PD-L1 expression.

### MAFG and NRF1 are required for super-enhancer- and LPS-induced PD-L1 expression

**Fig. 5:**
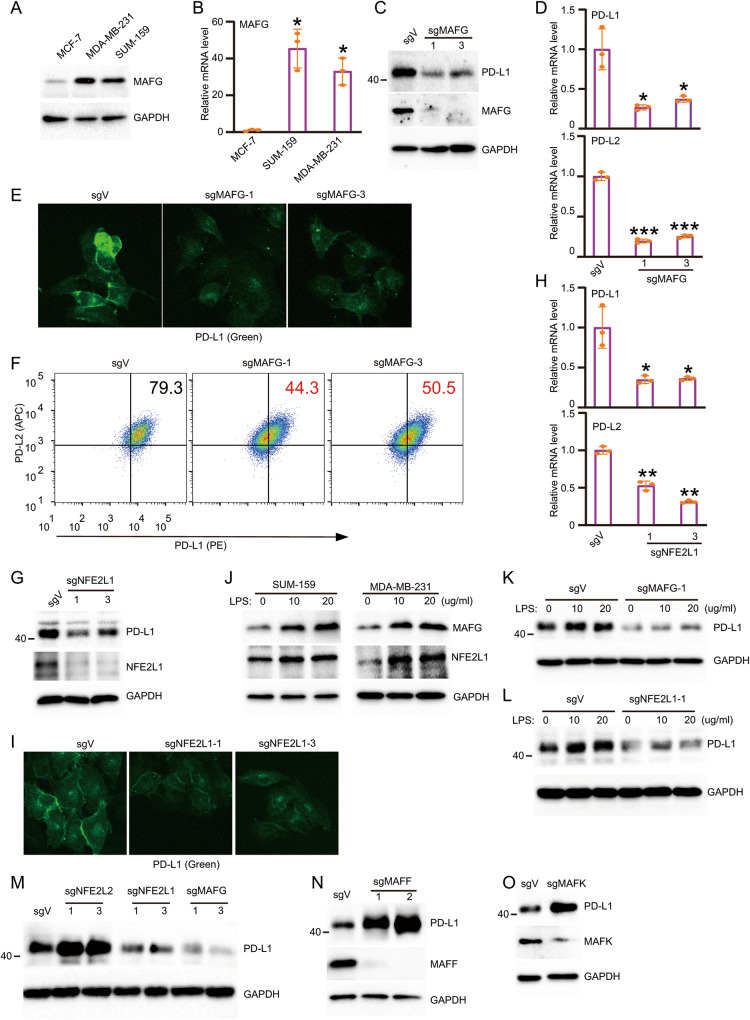
The transcription factors MAFG and NFE2L1 are required for super-enhancer- and LPS-induced PD-L1 expression. **A** Western blot analysis of the expression of MAFG in MCF-7, SUM-159 and MDA-MB-231 cell lines; **B** The relative mRNA level of MAFG was qualified by real-time PCR in MCF-7, SUM-159 and MDA-MB-231 cell lines; **C** Western blot analysis of PD-L1 and MAFG in SUM-159 that stably transfected with control and sgRNAs targeting on MAFG; **D** The relative mRNA levels of PD-L1 and PD-L2 in SUM-159 that stably transfected with control and sgRNAs targeting on MAFG; **E** Immunofluorescence assays were used to determine the PD-L1 expression in MAFG-deficient cells; **F** The surface expression of PD-L1(PE) and PD-L2(APC) in control and MAFG-deficient cells was determined by FACS; **G** Western blot analysis of PD-L1 and NFE2L1 in SUM-159 that stably transfected with control and sgRNAs targeting on NFE2L1; **H** The relative mRNA levels of PD-L1 and PD-L2 in control and NFE2L1 deficient cells was determined by RT-PCR; **I** Immunofluorescence assays were used to identify PD-L1 expression in NFE2L1 deficient cells; **J** SUM-159 and MDA-MB-231 were treated with LPS in indicated doses and the level of MAFG and NFE2L1 was measured by Western blot. **K** Control and MAFG-deficient cells were treated by LPS for indicate doses. The level of PD-L1 in these cells was examined by Western blot; **L** Control and NFE2L1 deficient cells were treated by LPS for indicated doses and the level of PD-L1 was determined by Western blot. **M** The protein level of PD-L1 in sgV, sgNFE2L2, sgNFE2L1 and sgMAFG were analyzed by WB. **N** The protein level of PD-L1 in sgV and sgMAFF cells were determined by WB. **O** The protein level of PD-L1 in sgV and sgMAFK cells were determined by WB. Data in (**A**–**D**, **G**, **H**, **J** and **K**) are representative of three independent experiments. Data in (**E**, **F**, **I**, **M**–**O**) are representative of two independent experiments. *p < 0.05; **p < 0.01, ***p < 0.001.

Because of the unexpected role of NRF2 in the expression of PD-L1, we examined whether other members instead of NRF2 in the NFE2:MAF family play a critical role in PD-L1 expression. We examined the expression of MAFG in MCF-7, SUM-159, and MDA-MB-231 cells. The expression of MAFG in SUM-159 and MDA-MB-231 cells is extremely higher than in MCF-7 cells (Fig. 5A, B). We established the MAFG knockout cells and found that the expression of PD-L1 was greatly reduced in MAFG-deficient cells (Fig. 5C). RT-PCR further confirmed that the transcription of PD-L1 and PD-L2 was greatly reduced upon knockout of MAFG (Fig. 5D). Consistently, immunofluorescence and flow cytometry indicated a significant reduction of PD-L1 in MAFG-deficient cells (Fig. 5E, F). Therefore, our results demonstrate the essential role of MAFG in super-enhancer-induced PD-L1 and PD-L2 expression. Next, we examined the role of NFE2L1 (NRF1) in the expression of PD-L1. Knockout of NFE2L1 significantly reduces the expression of PD-L1 (Fig. 5G). RT-PCR and immunofluorescence also indicated a significant reduction of PD-L1 upon genetic disruption of NFE2L1 (Fig. 5H, I). Taken together, MAFG and NRF1 are required for super-enhancer-induced PD-L1 expression. As LPS-induced PD-L1 expression relies on the super-enhancer, we determine whether MAFG and NRF1 are also required for LPS-induced PD-L1 expression. Upon LPS treatment, the expression of MAFG and NRF1 was significantly upregulated (Fig. 5J). In MAFG- and NRF1-deficient cells, LPS failed to induce the expression of PD-L1 (Fig. 5K, L). To further confirm the role of MAFG, NRF1 and NRF2 in the regulation of PD-L1, we compared the expression of PD-L1 in their deficient cells. Consistently, the expression of PD-L1 was reduced in MAFG and NRF1-deficient cells, but it was increased in NRF2-deficient cells (Fig. 5M). In addition, we also established the MAFF and MAFK deficient cells. The level of PD-L1 in MAFF and MAFK deficient cells was upregulated (Fig. 5N, O). Together, our results demonstrate the critical role of MAFG and NRF1 in both super-enhancer- and LPS-induced PD-L1 expression.

### Locus 22 affects BRD4 binding and loop formation but not H3K27Ac modification around the PD-L1L2-SE super-enhancer

**Fig. 6:**
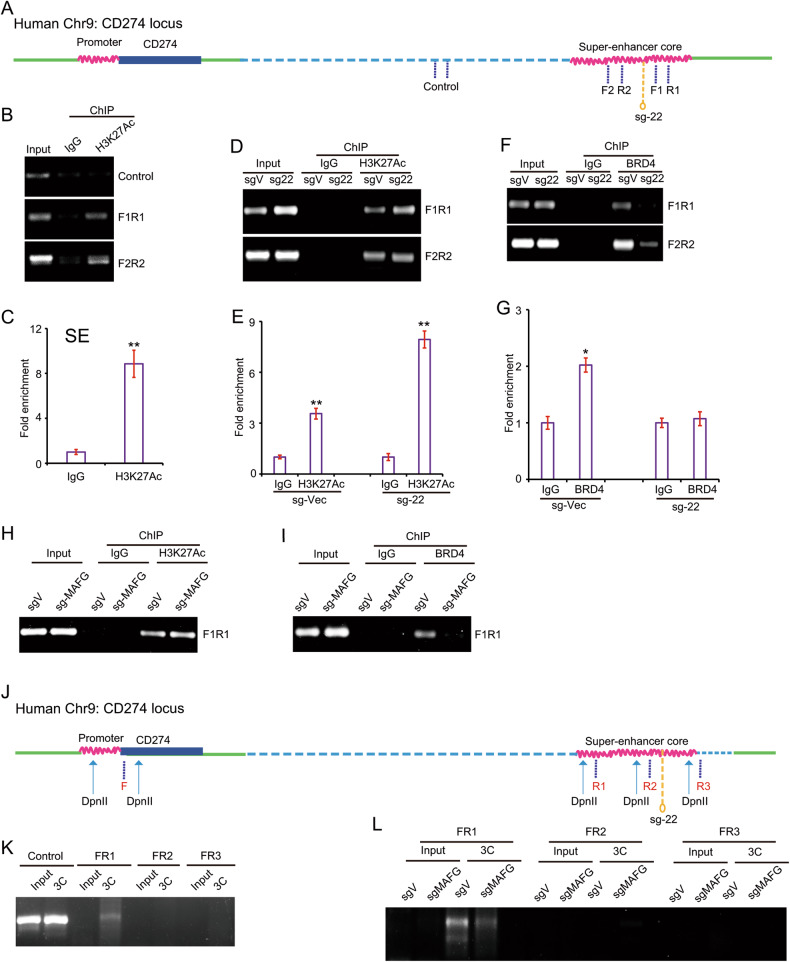
The locus 22 and its resident transcription factor MAFG affects BRD4 binding and loop formation but not histone H3K27Ac modification. **A** Schematic representation of the genomic locations of super-enhancer and promoter of *CD274* and the primers for ChIP-PCR; **B** The chromatin immunoprecipitation (ChIP) was performed by anti-H3K27Ac and control antibodies. Then the DNA in this samples were amplified by indicated primers and analyzed by agarose gel electrophoresis; **C** The amount of targeted DNA after ChIP was quantified by real-time PCR; **D** The histone modification of H3K27Ac within super-enhancer region in control and sg-22 cells was determined by ChIP-PCR; **E** The histone modification of H3K27Ac within super-enhancer region in control and sg-22 cells was quantified by real-time PCR; **F** The BRD4 binding within super-enhancer region in control and sg-22 cells was determined by ChIP-PCR; **G** The BRD4 binding within super-enhancer region in control and sg-22 cells was quantified by real-time PCR; **H** The histone modification of H3K27Ac within super-enhancer region in control and MAFG -deficient SUM159 cells was determined by ChIP-PCR; **I** The BRD4 binding within super-enhancer region in control and MAFG-deficient SUM159 cells was determined by ChIP-PCR; **J** The designed primers and DpnII digestion in PD-L1 promoter and PD-L1L2-SE super-enhancer region; **K** The interaction between the PD-L1L2-SE super-enhancer and the promoter region of PD-L1 was identified by 3C assay in SUM-159 cells; **L** The interaction frequency between the PD-L1L2-SE super-enhancer and the promoter region of PD-L1 was identified by 3C assay in control and MAFG-deficient SUM-159 cells. Data from (**B**, **D**, **F**, **H** and **I**) are representative of three independent experiments. Data from (**K**) and (**L**) are representative of two independent experiments. *p < 0.05; **p < 0.01, ***p < 0.001.

To understand how locus 22 regulates the expression of PD-L1, we performed ChIP-PCR to examine the histone modification of H3K27Ac in the core region of the super-enhancer (Fig. 6A). We found significant enrichment of H3K27Ac modification within super-enhancer but not control regions (Fig. 6B, C). Gene editing of locus 22 does not affect H3K27Ac (Fig. 6D, E). However, the BRD4 binding within the super-enhancer was greatly reduced after editing locus 22 (Fig. 6F, G). These results suggest that locus 22 is critical for BRD4 binding but does not influence H3K27Ac modification. Consistent with these results, knockout MAFG also greatly reduces the BRD4 binding with super-enhancer but has no effect on H3K27Ac (Fig. 6H, I). These results collectively demonstrate the critical role of MAFG in the BRD4 binding around the super-enhancer. The loop formation between the promoter and super-enhancer is critical for super-enhancer-induced gene expression. Thus, we examined whether MAFG is critical for loop formation. We performed chromatin conformation capture using *Dpn*II digestion (Fig. 6J). In control cells, we obtained clear PCR products using F and R1 primers, which suggests a loop formation between the super-enhancer and promoter (Fig. 6K). However, in MAFG knockout cells, the PCR product was greatly reduced, indicating a reduced interaction between the promoter and super-enhancer (Fig. 6L). Together, our results demonstrate the critical role of locus 22 and MAFG in BRD4 binding and loop formation.

### Locus 22 and its binding transcription factor NFE2L1/MAFG are required for immune evasion

**Fig. 7:**
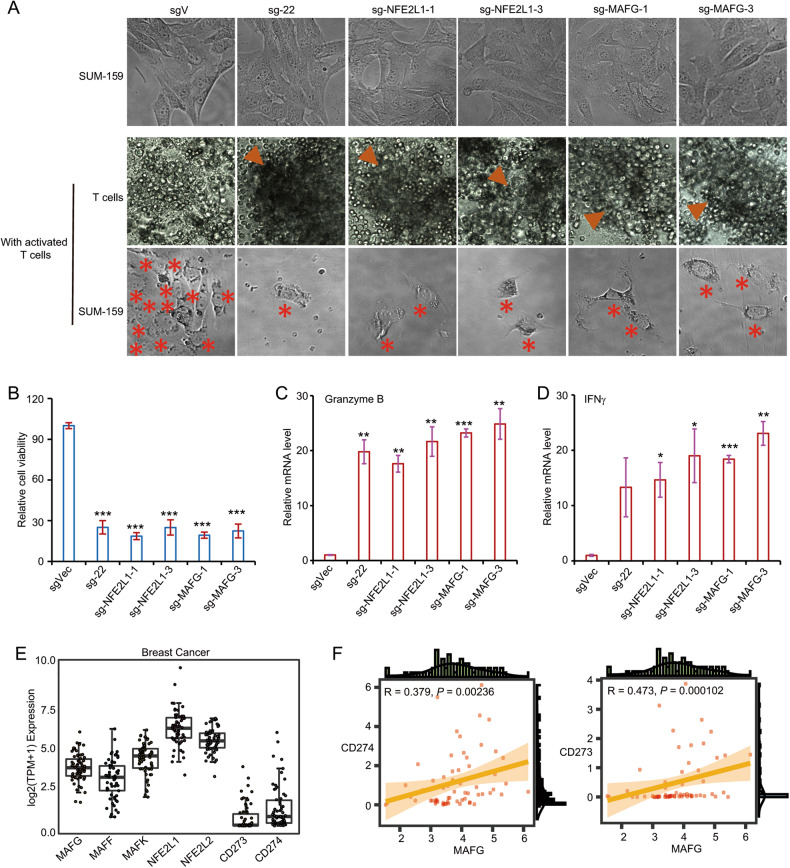
Cells with genetic modification of locus 22 and silence of MAFG or NEF2L1 are failed to escape the T cell-mediated killing. **A** Representative images of SUM-159 cell before and after co-cultured with activated T cells. Representative images of activated T cells co-cultured with SUM-159 cells or genetic modified cells (sgVector, sg-22, sg-MAFG-1, sg-MAFG-2, sg-NFE2L1-1 and sg-NFE2L1-2; **B** Quantification of remaining SUM-159 cells and its genetic modified cells after co-cultured with activated T cells; **C** Real-time PCR was performed to examine the relative mRNA levels of Granzyme B in activated T cells co-cultured with control or genetic modified cells; **D** Real-time PCR was performed to examine the relative mRNA levels of IFNγ in activated T cells co-cultured with control or genetic modified cells; **E** The expression of MAFG, MAFF, MAFK, NFE2L1, NFE2L2, CD273 and CD274 in breast cancer cell lines. **F** The expression of MAFG is positively correlated with PD-L1 and PD-L2 in breast cancer cell lines. Data from (**A**) and (**B**) are representative of three independent experiments. Data from (**C**) and (**D**) are representative of two independent experiments. *p < 0.05; **p < 0.01, ***p < 0.001.

Because of the critical role of locus 22 and NFE2L1/MAFG in the expression of PD-L1, we investigated whether locus 22 and NFE2L1/MAFG are critical for immune evasion. We co-cultured the SUM-159 cells with activated T cells. Even in the presence of activated T cells, the majority of SUM-159 cells survive, and the activated T cells cannot proliferate satisfactorily (Fig. 7A). However, the activated T cells proliferate and colonize satisfactorily when co-cultured with sg-22 cells (Fig. 7A). Very few sg-22 cells can survive in the presence of activated T cells. Furthermore, MAFG- and NRF1-deficient cells are also sensitive to T cell-mediated killing (Fig. 7A). Compared with control cells, sg-22 cells and NFE2L1/MAFG-deficient cells are sensitive to T cell-mediated killing (Fig. 7B). Consistently, T cells co-cultured with sg-22, sgMAFG, and sgNRF1 cells proliferate satisfactorily and produce much higher levels of Granzyme B and IFN-γ when compared with T cells co-cultured with control cells (Fig. 7C, D). These results demonstrate the critical role of locus 22, MAFG, and NRF1 in resistance to T-cell killing.

To further support these conclusions, we analyzed the cancer cell line database across all members of the NFE2:MAF family including MAFG, MAFF, MAFK, NFE2L1, and NFE2L2. In breast cancer cell lines, all five members exhibited relatively high expression (Fig. 7E). Across breast cancer cell lines, MAFG exhibited a strong correlation with PD-L1 (Fig. 7F). MAFG is also positively correlated with PD-L2 in breast cancer. Taken together, our results demonstrate the critical role of locus 22, NFE2L1, and MAFG in helping cancer cells evade T-cell killing by upregulating super-enhancer–mediated PD-L1 expression.

## Discussion

The precise functional engagement between PD-L1/L2 and PD-1 is critical for immune homeostasis. This achievement is partially caused by fine transcriptional regulation of PD-L1/L2. Although transcriptional regulation of PD-L1/L2 has been extensively studied, almost all of the investigations have been focused on the promoter region of CD274. Previously, we have identified a super-enhancer (PD-L1L2-SE) that is essential for the expression of PD-L1 and PD-L2. However, how the PD-L1L2-SE regulates PD-L1 and PD-L2 is largely undetermined. Here, by saturated CRISPR-Cas9 screening, we revealed that locus 22 and its binding transcription factor MAFG are required for the expression of PD-L1 and PD-L2. Locus 22 and binding of MAFG do not affect histone modification (H3K27Ac) but profoundly influence BRD4 binding and loop formation. More importantly, LPS as the major component of intratumoral bacteria can strongly induce PD-L1 expression. LPS-induced PD-L1 expression is completely dependent on the super-enhancer (PD-L1L2-SE) and its core DNA region. LPS-induced PD-L1 expression is also partially dependent on locus 22 and its binding transcription factor MAFG. Our findings provide new insight into understanding how locus 22 in the PD-L1L2-SE super-enhancer regulates the transcription of PD-L1. Intratumoral bacteria activate the PD-L1 relying on the super-enhancer, and this might greatly contribute to immune evasion and resistance to immune checkpoint blockade.

The small Maf (musculoaponeurotic fibrosarcoma) transcription factors including MAFG, MAFF, and MAFK were isolated two decades ago [26, 27]. The feature of sMAFs is that they harbor a basic region for DNA binding and a leucine zipper structure for dimer formation [28]. Because sMAFs lack any transcriptional activation domains, their function relies on homodimers or heterodimers with CNC transcription factors such as NRF1, NRF2, NRF3, Bach1, and Bach2 [29–31]. Genetic studies using NRF1 knockout mice suggested that the NRF1/MAFG heterodimer regulates genes associated with fatty acid metabolism and proteasome subunit genes [32]. In contrast, the NRF2/MAFG heterodimer regulates genes that are involved in oxidative response and glucose metabolism [33, 34]. In this study, we found that NRF2 negatively regulates PD-L1 expression, whereas NRF1 positively regulates PD-L1. This suggests that the NRF1/MAFG heterodimer instead of the NRF2/MAFG heterodimer plays a critical role in the regulation of super-enhancer- or LPS-induced PD-L1 expression. Recently, several groups have demonstrated the critical role of NRF2 in ultraviolet radiation- and interferon-γ-induced upregulation of PD-L1 [18, 19]. Thus, it is likely that different stimulations induce PD-L1 through either NRF2 or NRF1. However, the function of NRF1, NRF2, MAFG, MAFK, and MAFF in the regulation of PD-L1 under different conditions should be further explored extensively.

Intratumoral bacteria were first detected in certain tumor types a long time ago. However, recent approaches by bioinformatics mining and biological experiments demonstrate that intratumoral bacteria are not only frequently observed across cancer types but also associated with patient outcomes [20, 21, 35, 36]. Instead of melanomas, which were positive for lipoteichoic acid, the majority of other tumor types were positive for lipopolysaccharide [20]. However, it is still largely undetermined how intratumoral bacteria and their components, such as LPS, affect tumor development and response to therapy. Here, we found that LPS are a powerful inducer for PD-L1 transcription. Even in cells with the super-enhancer-derived high expression of PD-L1, LPS still strongly induce PD-L1 transcription. Thus, it is very likely that intratumoral bacteria will promote immune evasion by upregulation of PD-L1. Elimination of intratumoral bacteria might be beneficial to immune checkpoint blockade. However, the gut microbiome also has a profound effect on immune checkpoint blockade [37–39]. Thus, the specific elimination of intratumoral bacteria while maintaining the gut microbiome intact may enhance the therapeutic efficiency of immune checkpoint blockade. Furthermore, we found that LPS induce PD-L1 via transcription factor NFE2L1/MAFG. This conclusion is supported by genetic disruption of NFE2L1 and MAFG as well as NFE2/MAF inhibitor, ML385. ML385 is an inhibitor that binds the bZIP domain, which is a common domain that is present in other proteins, including NRF2, NRF1, MAFG, MAFK, and MAFF [40]. The specificity of ML385 to bZIP domain-containing proteins should be further explored. The detailed mechanism regarding NRF1/MAFG-mediated super-enhancer activation and intratumoral bacteria- or LPS-induced PD-L1 should be further explored.

In summary, we identified a genetic locus in the PD-L1L2-SE super-enhancer, and its resident transcription factor, MAFG, is critical for PD-L1 expression. Furthermore, the major component of intratumoral bacteria, LPS, could strongly induce PD-L1 expression. LPS-induced PD-L1 expression is completely dependent on the super-enhancer and partially dependent on locus 22. Our findings provide new insight into understanding the transcriptional regulation of PD-L1 and the critical role of MAFG in the regulation of PD-L1 and PD-L1-mediated immune evasion.

## Materials and methods

### Cell culture and treatment

Cell lines including SUM-159, MDA-MB-231 and MCF-7 were cultured in DMEM (cytiva) supplemented with 10% of FBS (allBio) and 1% of antibiotic mixture. Purified human T cells were maintained in RPMI 1640 medium (cytiva) supplemented with 10% of FBS (allBio) and 1% of antibiotic mixture. They were maintained at 37 °C under a humidified atmosphere with 5% of CO_2_. Before treatment with inhibitors, cell lines were implanted into 6-well plates at 24 h ago.

### Plasmids and transfections

We constructed sg-1, sg-2, sg-3, sg-4, sg-5, sg-6, sg-7, sg-8 sg-9, sg-10, sg-11, sg-12, sg-13, sg-14, sg-15, sg-16, sg-17, sg-18, sg-19, sg-20, sg-21, sg-22, sgNFE2L2-1, sgNEF2L2-2, sgMAFG-1, sgMAFG-2, sgNFE2L1-1 and sgNFE2L1-1 by ligation and digestion. The targeting oligonucleotides were listed at Supplementary Table S1 and were cloned into the epiCRISPR vector as following. The vector plasmids were treated with BSPQI enzyme (New England Biolads) at 50 °C for 4 h. The upstream and downstream primers were annealed at 94 °C for 20 min then cooling at room temperature for 1 h. The ligation was performed by using T4 DNA ligase (New England Biolads) at 16 °C overnight. Plasmids as mentioned above were validated by DNA sequencing. SUM-159 and MDA-MB-231 were transfected with different plasmids using Lipo2000 (invitrogen). After 48 h of transfection, the medium were replaced by complete medium containing 2 ug/ml puromycin (InvivoGen) for 7 days to select stable cell lines. All stable cell lines were examined by WB.

### Quantitative real-time RT-PCR

Total RNA was extracted from cells by TRIzol RNA isolation reagents (Invitrogen) and reversely transcripted to cDNA by HiScript® II QRT SuperMix for qPCR (+gDNA wiper) kit (Vazyme). The expression of targeted gene was detected by AceQ® qPCR SYBR Green Master Mix kit (Vazyme) according to confirmed instruction using CFX96 real-time PCR system (Bio Rad). Fold changes in gene expression were calculated using 2−△△t method and normalized to the expression of GAPDH. The primers are listed in Supplementary Table S2.

### Western blotting

Total protein was extracted from cells by RIPA buffer (Solarbio) containing 1% PMSF (Solarbio). Protein samples were separated using SDS-PAGE gels of different concentration according to various sizes. PVDF membrane (Millipore) was used to transfer proteins from gels and then blocked for 2 h at room temperature using 5% of non-fat milk. The membrane were incubated with probed primary antibodies including: anti-PD-L1 (Abcam,1:1000), anti-NRF2 (Abclonal,1:500), anti-MAFG (Abclonal,1:500), anti-NFE2L1 (Abclonal,1:500), anti-GAPDH (Santa Cruz Biotechnology,1:2000), anti-HSP70 (Santa Cruz Biotechnology,1:2000), anti-HSP90 (Santa Cruz Biotechnology,1:2000) at 4 °C overnight. Then, the membranes were incubated with the peroxidase-conjugated secondary antibody for 1 h at room temperature. The blots were visualized using an ECL kit (Biosharp) and analyzed by ImageJ system.

### Immunofluorescence

Different cells were planted into the round glass slide in 24-wells plate at the same density and fixed with 4% paraformaldehyde for 20 min at room temperature. Then the samples were washed with PBS and blocked by 3% BSA for 2 h at room temperature. Then the primary antibody including anti-PD-L1(Abcam,1:500) was incubated with the samples at 4 °C overnight. After washing 3 times for 5 min each, secondary antibody including Alexa Fluor 488 conjugated anti-rabbit (Beyotime,1:500) was added and incubated overnight. Next, Hoechst dye (Beyotime,1:500) was used in nuclear staining for 20 min at room temperature. Finally, the fluorescent microscope (Leica) was applied to detect Fluorescence signals.

### Flow cytometry

Cells were seeded into 6-well plates at the same density in advance. Next day, cells were collected and washed with PBS for 3 times and stained with antibodies including anti-PD-L1 (PE conjugated) (Elabscience, E-AB-F1133D) and anti-PD-L2 (APC conjugated) (Elabscience, E-AB-F1175E) in staining buffer for 40 min. After that, samples were centrifuged for 3 min and washed with 3 times, then suspended with PBS. The signals were captured by a BD Calibur (BD Biosciences) flow cytometer and analyzed by FlowJo software.

### T cell-mediated tumor cell killing assay

CD8+ T cells or CD3+ T cells were selected by FACS or anti-FITC microbeads from human peripheral blood mononuclear cells (PBMCs). The human PBMCs were separated from the whole blood by a density gradient centrifugation using Ficoll-Paque solution. Isolated T cells were activated by anti-CD3 antibodies, anti-CD28 antibodies and IL-2 cytokine. SUM-159 cells and its genetic modified cell lines were seeded into the 96-well plate. In some experiments, seeded SUM-159 cells were also treated with inhibitors before co-culture with activated T cells. The activated T cells were incubated with SUM-159 cells at ratio of 1:10 (cancer cells: activated T cells). Forty-eight hours after incubation, the T cells were washed by PBS for extraction of total RNA. The survived cancer cells in the bottom were photographed and quantified by CCK-8 kit. The ethics committees of the Nantong University approved the study protocol and the healthy donor provided written informed consent.

### ChIP (chromatin immunoprecipitation)-PCR

ChIP was performed by a ChIP Assay Kit (Beyotime) according to the instructions. In brief, cells were cultured in 10 cm dishes at 24 h before and cross-linked by 1% formaldehyde for 10 min at room temperature. Then, glycine was added to terminate the cross-linking for 5 min. Cells were collected and sonicated to shear DNA to suitable length and incubated with 60 µL of protein A/G beads and 2 µg of primary antibody on a rotator at 4 °C overnight. Next, the DNA-protein complex was washed and eluted. The DNA was purified with a DNA Purify Kit (Beyotime) and detected by realtime PCR.

### Chromatin conformation capture (3C)

Samples were prepared the same as ChIP protocol. Nuclear were obtained and digested by DpnII at 37 °C overnight. Next, 20% SDS was added to inactivate the restriction enzymet at 65 °C for 30 min and T4 DNA ligase (Vazyme) was added for ligation at 16 °C for 4 h and at room temperature for 30 min. Then proteinase K was added to de-crosslink the sample at 65 °C overnight. Finally, the digested DNA was purified with a DNA Purify Kit (Beyotime) and detected by realtime PCR.

### Statistical analysis

To compare the control and knockout cells, the two-tailed Student’s *t* test was used to determine the statistical significance. In stimuli treatment experiments, the Two-tailed Student’s *t* test was used to analyze the effect. In correlation analysis, we conduct the correlation analysis and provide the *p* value as well as the Spearman Rho estimate. All data are presented as the mean ± standard deviation (SD). All statistical details and the times of biological repeats were provided in the figure legends. A value of *p* < 0.05 was considered statistically significant. *p < 0.05; **p < 0.01; ***p < 0.001.

### Supplementary information

Supplemental Tables

## Data Availability

All data are available upon reasonable request.
