# Supplementary material for: Identifying a locus in super-enhancer and its resident NFE2L1/MAFG as transcriptional factors that drive PD-L1 expression and immune evasion
Source: Oncogenesis. 2023 Nov 20;12(1):56. doi: 10.1038/s41389-023-00500-3 (PMC10662283; doi:10.1038/s41389-023-00500-3)
Supplement: Supplementary file 1 — Supplemental Tables [file 41389_2023_500_MOESM1_ESM.docx]

**Identifying a locus in super-enhancer and its resident NFE2L1/MAFG as transcriptional factors that drive PD-L1 expression and immune evasion**

Conglin Shi ^1, 2^, Liuting Chen ^1^, Hui Pi ^3^, Henglu Cui ^1, 2^, Chenyang Fan ^1, 2^, , Fangzheng Tang ^4^, Xuanhao Qu ^2^, Rong Sun ^2^, Fengbo Zhao ^2^，Yihua Song ^5^, Yuanyuan Wu ^2^, Miaomiao Chen ^2^, Wenkai Ni ^6^, Lishuai Qu ^6^, Renfang Mao ^3, *^, Yihui Fan ^1, 2, *^

**Supplementary Table S1: List of RT-PCR Primers**

| Name | sense | antisense |
| --- | --- | --- |
| Human-18S | GAACGAGACTCTGGCATGCTA | CACGCTGAGCCAGTCAGTGTA |
| Human-β-Actin | TTGTTACAGGAAGTCCCTTGCC | ATGCTATCACCTCCCCTGTGTG |
| Human-PD-L1 | GTAGCACTGACATTCATCTTC | TTCCTTCCTCTTGTCACGCTC |
| Human-PD-L2 | CATAGCCACAGTGATAGCCCT | GGCTCCCAAGACCACAGGTTC |
| Human-MAFG | AGAGCGCCTGCTCGCTGTGCC | GTCACCAGCTCCTCATCCGTC |
| Human-IFNγ | GGTTCTCTTGGCTGTTACTG | ATCCGCTACATCTGAATGAC |
| Human-Granzyme B | GACAGTACCATTGAGTTGTGC | CTGGGCCACCTTGTTACACAC |
|  |  |  |

| **Supplementary Table S2: List of oligo sequences for sgRNAs**   \| Name \| Oligo sequence \| \| --- \| --- \| \| Sg-9 \| CCAAGCAGAGTGGGAAATAC \| \| Sg-10 \| TTGCATTACACTGCATTTGG \| \| Sg-11 \| GGAAAATATCTTGGGAGGAA \| \| Sg-12 \| CTGTGAATGTAGGGCAGAGT \| \| Sg-13 \| AAGATCACCCTGTGAATGTA \| \| Sg-14 \| CTACTCTGCCCTACATTCAC \| \| Sg-15 \| CTACATTCACAGGGTGATCT \| \| Sg-16 \| GGCCCCTCCTATCTTATAGA \| \| Sg-17 \| TTTCCCTTCTATAAGATAGG \| \| Sg-18 \| ATGTCACCAGCCAATCTATG \| \| Sg-19 \| GAGGGGCCTCATAGATTGGC \| \| Sg-20 \| TTCAACATTGCATTGTCACT \| \| Sg-21 \| AGTGACAATGCAATGTTGAA \| \| Sg-22 \| AAGTGACAATGCAATGTTGA \| \| Sg-NFE2L1-1 \| GGGCAGTGAAGTAATTGTCC \| \| Sg-NFE2L1-3 \| GCTGAGTTTGATTGGGGTAC \| \| Sg-MAFG-1 \| GCACCTGCGGGGCCTGTCCA \| \| Sg-MAFG-3 \| GAGCTGAACCAGCACCTGCG \| \| Sg-NFE2L2-1 \| GGTTTCTGACTGGATGTGCT \| \| Sg-NFE2L2-2 \| GGACATTGAGCAAGTTTGGG \| \| Sg-NFE2L2-3 \| GGCTTCTGGACTTGGAACCA \| |  |
| --- | --- | --- | --- | --- | --- | --- | --- | --- | --- | --- | --- | --- | --- | --- | --- | --- | --- | --- | --- | --- | --- | --- | --- | --- | --- | --- | --- | --- | --- | --- | --- | --- | --- | --- | --- | --- | --- | --- | --- | --- | --- | --- | --- | --- | --- |
